# LIN28B is highly expressed in atypical teratoid/rhabdoid tumor (AT/RT) and suppressed through the restoration of SMARCB1

**DOI:** 10.1186/s12935-016-0307-4

**Published:** 2016-04-18

**Authors:** Seung Ah Choi, Seung-Ki Kim, Ji Yeoun Lee, Kyu-Chang Wang, Chanhee Lee, Ji Hoon Phi

**Affiliations:** Division of Pediatric Neurosurgery, Seoul National University Children’s Hospital, 101 Daehakro, Jongno-gu, Seoul, 03080 Republic of Korea; Department of Anatomy, Seoul National University College of Medicine, Seoul, 03080 Republic of Korea

**Keywords:** Atypical teratoid/rhabdoid tumor, LIN28B, SMARCB1

## Abstract

**Background:**

Atypical teratoid/rhabdoid tumor (AT/RT) is a highly malignant brain tumor that almost exclusively develops in young children. AT/RT belongs to the embryonal brain tumor group, comprising primitive tumors recapitulating the early development of the central nervous system during embryogenesis. The loss of SMARCB1 protein expression is a hallmark of AT/RT pathogenesis. LIN28A/B is a key gene in embryonic development and for the maintenance of pluripotency in stem cells. LIN28B might be an important co-player in AT/RT pathogenesis, considering the primitive nature and young age onset of AT/RT.

**Methods:**

We explored the expression patterns of LIN28B in AT/RT and compared it with the expression in cortical dysplasia and medulloblastoma. The functional role of LIN28B was assessed using LIN28B-siRNAs in primary cultured AT/RT cells.

**Results:**

LIN28B is highly expressed in AT/RT compared with medulloblastoma and other embryonal tumors, whereas primary let-7g miRNA is down-regulated. AT/RT also showed higher expression of CCND1 and MYC, and lower expression of CDKN1C. The suppression of CCND1 expression and enhanced expression of CDKN1C were also observed. The knockdown of LIN28B decreased cell viability and proliferation, induced cell cycle arrest, and reduced migration in primary cultured AT/RT cells. Furthermore, we showed that the knockdown of LIN28B decreased the expression of other pluripotency-related genes (OCT4 and NANOG) and the mesenchymal-epithelial transition signature. We also transfected wild-type SMARCB1 into primary cultured AT/RT cells. The restoration of SMARCB1 in AT/RT cells decreased the expression of LIN28B and CCND1.

**Conclusions:**

These results show that LIN28B might be regulated through SMARCB1; the loss of SMARCB1 protein in AT/RT results in the unopposed expression of LIN28B and related oncogenes such as CCND1, leading to tumorigenesis. Therefore, the strategic role of LIN28B in AT/RT might be utilized as an important therapeutic target.

**Electronic supplementary material:**

The online version of this article (doi:10.1186/s12935-016-0307-4) contains supplementary material, which is available to authorized users.

## Background

Atypical teratoid/rhabdoid tumor (AT/RT) is a highly malignant brain tumor that predominantly develops in children. Despite dramatic improvements in treatments for other pediatric cancers, currently the prognosis of patients with AT/RT is dismal, with less than 10–20 % of patients attaining survival for more than 2 years [1]. Deletions and mutations of the SMARCB1 (BAF47/SMARCB1/SNF5) gene are hallmarks of AT/RT tumors. SMARCB1 is a component of the SWI/SNF chromatin remodeling complex, and the loss of SMARCB1 function affects thousands of genes across the genome [2].

The biology contributing to the aggressiveness of AT/RT remains elusive. In a previous study, we observed that AT/RT brain tumor-initiating cells showed the robust expression of LIN28A/B when compared with other brain tumors, such as medulloblastoma and glioblastoma [3]. Additionally, recent studies have demonstrated that LIN28A/B are highly expressed in AT/RT primary tumors and cell lines, and the knockdown of LIN28A suppresses AT/RT growth and tumorigenicity [4]. The activation of LIN28A/B occurs in several different primary human tumors and plays an important role in cancer progression and metastasis [5, 6], involving stemness [7] through the negative regulation of the maturation of let-7 microRNA (miRNA) family members [8].

In the present study, we focused on LIN28B/let-7g and CCND1 for several reasons. First, LIN28B overexpression in human cancers has been frequently associated with human tumorigenesis compared with LIN28A [9, 10]. Second, several studies have demonstrated that LIN28B is a cytoplasmic protein shuttled into the nucleus in a cell-cycle dependent manner [5, 11, 12]. Third, a recent study showed that LIN28B promoted colon cancer migration and recurrence [8]. Human LIN28B recognizes let-7g [13]. We reported reciprocal expression between LIN28 and miRNA let-7g in AT/RT cells in a previous study [14].

In the present study, the expression and functional role of LIN28B in AT/RT was addressed. The inhibition of LIN28B expression increased let-7g expression, down-regulated CCND1 and up-regulated CDKN1C. The knockdown of LIN28B decreased the proliferation and migration of AT/RT cells. Furthermore, the knockdown of LIN28B affected the expression of pluripotency- and mesenchymal-epithelial transition (EMT)-related genes. As the loss of function of SMARCB1 is a genetic hallmark of AT/RT, we explored the relationship of SMARCB1 and LIN28B. The restoration of SMARCB1 expression in AT/RT cells suppressed LIN28B over expression and decreased cell proliferation.

These results indicate that LIN28B/let-7g/CCND1 is a key factor in AT/RT tumorigenesis, and the loss of SMARCB1 leads to LIN28B overexpression.

## Methods

### Patients and tissue samples

Human brain tissues were collected from patients diagnosed with cortical dysplasia (CD; N = 4), medulloblastoma (MB; N = 8) and AT/RT (N = 10) who received surgery at the Seoul National University Children’s Hospital (Table 1). Eligible patients and/or their parents provided written informed consent to donate tumor tissue samples. The Institutional Review Board (IRB) of the Seoul National University Hospital approved the tissue banking and study protocols (IRB # 1406-044-584). None of the patients received neo-adjuvant therapies. Tissue selection was determined based on tissue availability and a person blinded to the treatment outcome of the patients performed the selection.

**Table 1:** Patient information

Designation	Sex	Age	Diagnosis	Frozen-mRNA/miRNA	Paraffin-IHC	Western blotting
C1	F	21 year	Cortical dysplasia	o	o	o
C2	F	16 year	Cortical dysplasia	o	o	o
C3	M	18 year	Cortical dysplasia	o	o	o
C4	M	14 year	Cortical dysplasia	o	–	–
M1	M	13 year	Medulloblastoma	o	o	o
M1	F	8 year	Medulloblastoma	o	o	o
M3	M	3 year	Medulloblastoma	o	o	o
M4	F	8 month	Medulloblastoma	o	o	o
M5	F	5 year	Medulloblastoma	o	o	o
M6	F	11 year	Medulloblastoma	o	–	o
M7	M	12 month	Medulloblastoma	o	–	–
M8	M	11 year	Medulloblastoma	o	–	–
A1	M	11 month	AT/RT	o	o	o
A2	F	13 month	AT/RT	o	o	o
A3	F	3 month	AT/RT	o	o	o
A4	M	1 year	AT/RT	o	o	o
A5	M	1 month	AT/RT	o	o	o
A6	M	12 month	AT/RT	o	–	o
A7	M	9 month	AT/RT	o	–	o
A8	M	13 month	AT/RT	o	–	o
A9	M	17 month	AT/RT	o	–	–
A10	M	1 month	AT/RT	o	–	–

The tissues used for each experiment are marked

*AT/RT* atypical teratoid rhabdoid tumor

### Primary cell culture

Fresh AT/RT (from a 1-month-old boy: SNU-AT/RT3 and a 13-month-old boy: SNU-AT/RT4), MB (from a 7-year-old boy) and glioblastoma (from a 43-year-old man) tissues were obtained and enzymatically dissociated into single cells as previously described [3]. The tumor cells were cultured in Dulbecco’s Modified Eagle’s Medium (DMEM; Life Technologies, Carlsbad, CA, USA) supplemented with 10 % fetal bovine serum (FBS; Life Technologies) and penicillin–streptomycin (Life Technologies). The primary cultured cells obtained from the AT/RT, MB and glioblastoma tissue samples were only used in early passages (<4) for experiments. The cells were incubated at 37 °C with 5 % CO_2_ in a humidified atmosphere.

### Real-time quantitative reverse transcription polymerase chain reaction (RT-qPCR)

Total RNA with miRNA was extracted from tissues (Table 1) and cells using a RNA isolation Kit (Life Technologies) according to the manufacturer’s instructions. Total RNA from the normal brain was purchased from Clontech Laboratories (Mountain View, CA, USA).The real-time RT-qPCR analysis of mRNA/mature miRNA was performed using the TaqMan mRNA or microRNA Assay Kit (Life Technologies) on an ABI 7000 system (Applied Biosystems, Foster City, CA, USA) according to the manufacturer’s instructions. TaqMan probes for LIN28A, LIN28B, SMARCB1, CCND1, CDKN1C, MYC, SOX2, OCT4, C-MYC, KLF4, glyceraldehyde 3-phosphate dehydrogenase (GAPDH), primary-let-7g (pri-let-7g), mature-let-7g and RUN6B were used. The reactions were performed under the conditions specified in the ABI TaqMan assay protocol. All reactions were repeated in triplicate, and the comparative threshold cycle (ΔCt) method was used to calculate the relative gene expression. The results were normalized to GAPDH for mRNA or RUN6B for miRNA and were presented relative to normal brain expression [15].

### Immunohistochemistry

Paraffin-embedded tissues were acquired from CD, MB and AT/RT patients (Table 1). Immunohistochemistry with anti-LIN28A, anti-LIN28B, anti-CCND1 and anti-CDKN1C antibodies (Abcam, Cambridge, MA, USA) was performed as previously described [15].

### siRNA transfections

For LIN28B knockdown analysis, negative siRNA and two different sequences for LIN28B siRNA (LIN28B siRNA-1 and LIN28B siRNA-2) were purchased from Bioneer (Daedeok-gu, Daejeon, Korea). The siRNA transfections were performed using Lipofectamine^®^ RNAiMAX (Life Technologies) according to the manufacturer’s instructions.

### SMARCB1 overexpression

To construct SMARCB1, full-length SMARCB1 was cloned into the mammalian expression vector pEGFP-C2 (Clontech, CA, USA). The SMARCB1 coding sequence was PCR amplified using the forward (5′-CCCGAAGCTTCATGATGATGGCGCTGAGCAAG-3′) and reverse (5′-CCGGAATTCTTACCAGGCCGGGCCCGTGTTGGCA-3′) primers containing *Hin*dIII and *Eco*RI restriction sites. The constructs were verified through sequencing.

To generate cells expressing pEGFP-C2.SMARCB1, SNU-AT/RT3 and SNU-AT/RT4 cells were transfected using the Neon Transfection System (Life Technologies) as previously described [16]. The electroporation conditions were 1400 V with a 20-pulse width and 1 pulse. The transfection efficiency was measured using a fluorescence microscope and confirmed in cell lysates using RT-PCR and western blotting.

### Western blotting

Total proteins were isolated from tissues and cells, the protein concentration was determined, and western blot analysis was performed as previously described [15]. Anti-LIN28A (1:200; Cell Signaling Technology, Danvers, MA, USA), anti-LIN28B (1:200; Cell Signaling Technology), anti-CCND1 (1:1000; Thermo Fisher Scientific, Fremont, CA, USA) anti-CDKN1C (1:1000; Novus Biologicals, Littleton, CO, USA), and anti-β-actin (1:5000; Sigma-Aldrich, St. Louis, MO, USA) antibodies were used.

### Cell viability and proliferation assay

After transfecting the LIN28B siRNAs, the cytotoxic effects were determined using colorimetric cell counting kit-8 (CCK-8; Dojindo Molecular Technologies, Rockville, MD, USA) and Roche Colorimetric Assay kit 1 (BrdU labeling and detection kit III; Roche Diagnostics GmbH, Germany) according to the manufacturer’s instructions. The absorbance of the samples against a background control was measured using a Microplate Reader (Molecular Devices, Sunnyvale, CA, USA) at a wavelength of 450 nm for CCK-8 and 575 nm for BrdU. The viability or proliferation of negative siRNA-treated cells was regarded as 100 %.

### Cell cycle analysis

The LIN28B siRNA-transfected cells were harvested and fixed in 70 % alcohol overnight at −20 °C. After washing with phosphate-buffered saline (PBS), the cells were incubated with 0.2 mg/ml RNase (Sigma, St. Louis, MO, USA) at 37 °C for 30 min and subsequently 10 µg/ml of propidium iodide was added. At least 20,000 stained cells were analyzed, and the percentages of cells in G0/G1, S and G2/M phases were determined using a FACS Calibur flow cytometer (Becton–Dickinson, Franklin Lakes, NJ, USA).

### Migration assay

After transfection, the cells were harvested in the serum-free medium and introduced into the upper chamber (8-μm pore size, Corning, NY, USA). Culture medium supplemented with 10 % FBS were added to the lower chambers as the chemoattractant. After 24 h, the migration assays were performed as previously described [15].

### Statistical analysis

All experiments are displayed as the mean ± standard deviation (SD) or expressed as a percentage of the controls ± SD. A two-tailed ANOVA assay and Student’s t test were used to determine the differences between data groups, and statistically significant differences were considered at p < 0.05. GraphPad Prism software (La Jolia, CA, USA) was used for all the analyses. All experiments were conducted in triplicate.

## Results

### Amplification of LIN28B in AT/RT

**Fig. 1:**
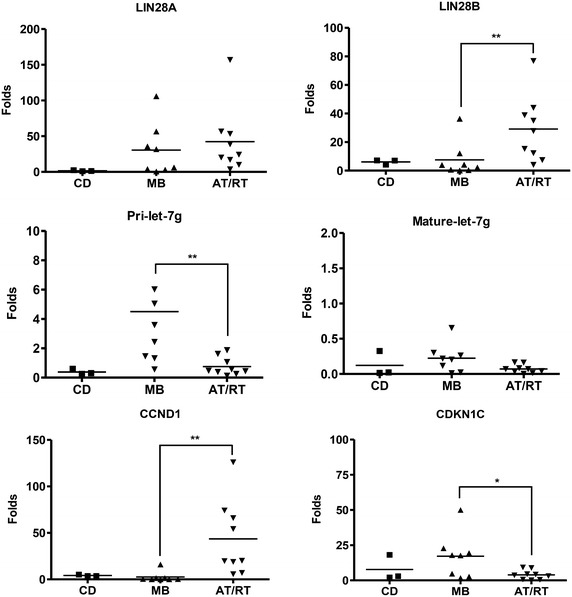
Relative expression of mRNAs and miRNAs in cortical dysplasia (CD), medulloblastoma (MB) and AT/RT clinical samples. The relative expression of LIN28A, LIN28B, pri-let-7g, mature let-7g, CCND1, CDKN1C and MYC was detected using RT-qPCR. The levels of Lin28B and CCND1were upregulated, while the levels ofpri-let-7g, and CDKN1C were significantly down-regulated in AT/RT. The expression of LIN28B, rather than LIN28A, is more represented in AT/RT compared with CD and MB. *P < 0.05; **P < 0.01. *Error bars* represent ±SD

**Fig. 2:**
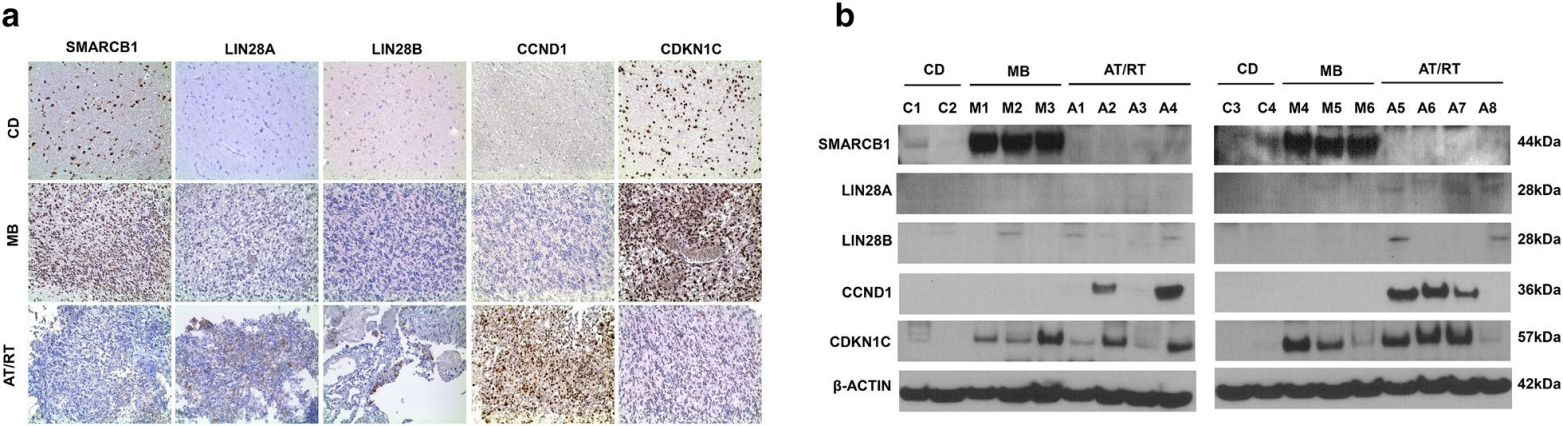
Comparison of the expression of LIN28A, LIN28B, SMARCB1, CCND1, and CDKN1C in CD, MB and AT/RT tissues. **a** Representative immunohistochemistry (IHC) images reveal the increased expression of LIN28A, LIN28B, and CCND1 and reduced expression of CDKN1C in AT/RT tissues. The loss of SMARCB1 expression was also observed in AT/RT, ×200. **b** The results of the western blot analysis were consistent with the IHC results

We investigated the relative levels of LIN28A, LIN28B, pri-let-7g, mature let-7g, CCND1, and CDKN1C expression using RT-qPCR. The relative expression of LIN28A (3.8–156.9-fold; p = 0.009), LIN28B (4.1–76.9-fold; p = 0.012) and CCND1 (5.7–126.0-fold; p = 0.009) were higher in AT/RT tissues, compared with cortical dysplasia tissues, (Fig. 1; Table 2). The expression of pri-let-7g (0.4–1.9-fold; p = 0.517), mature let-7g (0.0–0.3-fold; p = 1.000) and CDKN1C(0.2–9.1-fold; p = 0.864) were similar in AT/RT tissues. The expression of LIN28B (p = 0.008), CCND1 (p = 0.013), pri-let-7g (p = 0.007), and CDKN1C (p = 0.036) were significantly higher in AT/RT tissues than in MB tissues. This difference was not observed for LIN28A (p = 0.441) and mature-let-7g (p = 0.075). We next examined protein expression using immunochemistry and western blot analysis. The results of immunohistochemistry showed that LIN28B protein expression was predominantly detected in AT/RT in MB tissues compared with LIN28A (Fig. 2a). The CCND1 and CDKN1C expression patterns were mutually exclusive in AT/RT and MB tissues. To further confirm these findings, we performed western blot analysis. The expression patterns of LIN28A, LIN28B, CCND1 and CDKN1C were remarkably similar to the RT-qPCR and immunochemistry results (Fig. 2b). Taken together, these data show that the expression of LIN28B is more specific than LIN28A in AT/RT.

**Table 2:** RT-qPCR results for mRNA and miRNAs

Designation	LIN28A	LIN28B	CCND1	CDKN1C	Pri-let-7g	Mature let-7g
C1	1.2	7.1	5.1	18.1	0.3	0.0
C2	1.0	4.2	3.7	2.1	0.3	0.0
C3	2.5	7.0	3.7	2.9	0.6	0.3
C4	1.5	3.2	4.3	2.5	0.2	0.1
M1	0.6	4.2	1.5	22.8	15.6	0.2
M1	4.1	36.4	0.3	50.1	6.0	0.0
M3	6.3	12.1	1.2	17.7	3.6	0.3
M4	56.7	0.4	0.2	4.7	2.4	0.2
M5	3.1	0.7	1.5	1.8	0.6	0.0
M6	106.0	2.2	16.1	19.4	5.0	0.3
M7	35.8	0.1	0.2	18.0	1.5	0.1
M8	31.9	4.0	0.4	2.7	1.3	0.7
A1	156.9	35.0	7.0	0.2	0.4	0.2
A2	53.5	38.9	19.8	3.1	0.5	0.2
A3	20.2	12.3	66.1	8.9	1.9	0.0
A4	3.8	15.3	5.7	0.7	0.1	0.1
A5	17.0	76.9	74.2	4.3	1.1	0.0
A6	24.3	7.5	18.9	9.3	1.6	0.1
A7	38.8	27.8	126.0	3.7	0.4	0.0
A8	10.1	4.1	54.3	0.5	0.3	0.0
A9	56.7	44.0	20.0	4.5	0.6	0.1
A10	26.6	65.5	57.3	5.2	0.5	0.3

The values are normalized to GAPDH or RUN6B

### The knockdown of LIN28B in primary cultured AT/RT cells

**Fig. 3:**
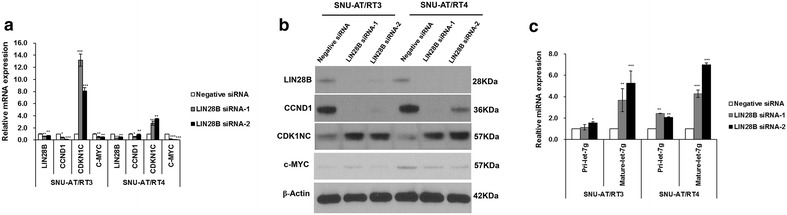
Effect of LIN28B knockdown on AT/RT cells. The level of LIN28B mRNA and protein was effectively inhibited after the transfection of LIN28B-siRNAs. **a** RT-qPCR and **b** western blot analysis showed that LIN28B knockdown reduced CCND1 expression and enhanced CDKN1C expression. **c** The increased expression of pre-leg-7g and mature let-7g miRNA was observed after LIN28B knockdown. *P < 0.05; **P < 0.01; ***P < 0.01. *Error bars* represent ±SD

To determine the role of LIN28B in AT/RT, we performed siRNA knockdown experiments using two distinct siRNA constructs (LIN28B siRNA-1 and LIN28B siRNA-2). The knockdown efficiency was confirmed using RT-qPCR and western blot analysis in two primary cultured AT/RT cell lines (SNU-AT/RT3 and SNU-AT/RT4). The LIN28B siRNAs effectively degraded LIN28B (LIN28B siRNA-1 in SNU-AT/RT3, p = 0.009; LIN28B siRNA-1 in SNU-AT/RT4, p = 0.002; LIN28B siRNA-2 in SNU-AT/RT3, p = 0.001; LIN28B siRNA-2 in SNU-AT/RT4, p = 0.02; Fig. 3a; Additional file 1: Table S1). The inhibition of LIN28B suppressed CCND1 expression but enhanced CDKN1C expression (Fig. 3a; Additional file 1: Table S1). Western blot analysis after LIN28B siRNA treatment further confirmed the RT-qPCR results (Fig. 3b; Additional file 1: Table S1). The increased expression of pri-let-7g and mature let-7g miRNA were observed after LIN28B knockdown (Fig. 3c; Additional file 1: Table S1).

### Functional roles of LIN28B

**Fig. 4:**
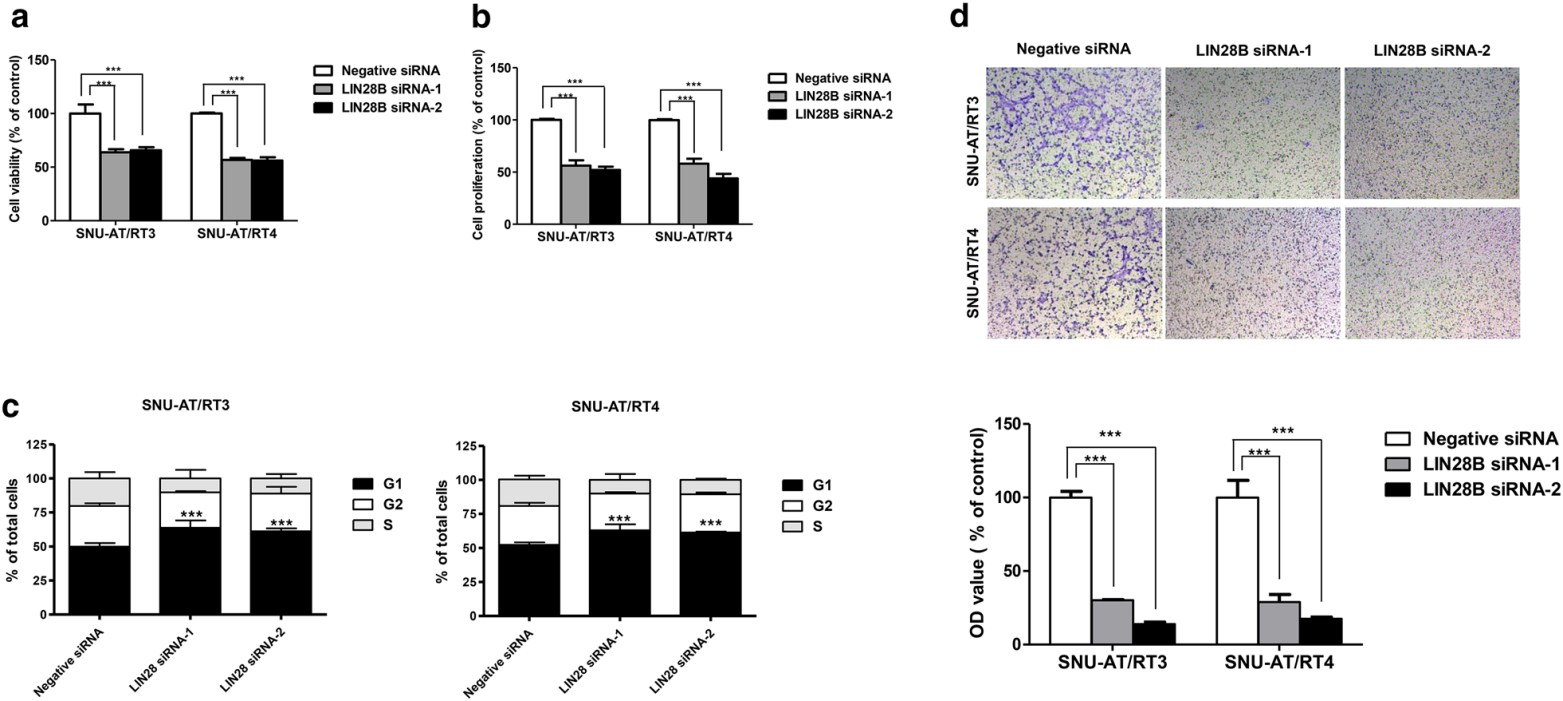
Functional studies after LIN28B knockdown on AT/RT cells in vitro.LIN28B knockdown impacts AT/RT cell growth and migration. **a**–**c** Representative histograms showed that LIN28B inhibition reduced cell viability and cell proliferation, inducing cell cycle G1 arrest. **d** Representative images and the quantification of migrated cells revealed the inhibition of AT/RT cell migration after LIN28B knockdown. ***P < 0.001. *Error bars* represent ±SD

To investigate the functional role of LIN28B, viability, proliferation, cell cycle, and migration of AT/RT cells were assessed after LIN28B knockdown. LIN28B knockdown in AT/RT cells resulted in a significant reduction of cell viability and proliferation (Fig. 4a, b). Cell viability was significantly reduced after LIN28 knockdown (negative siRNA vs. LIN28B siRNA-1 or LIN28B siRNA-2: 100 ± 8.5 vs. 63.8 ± 2.8 or 65.7 ± 2.8 % in SNU-AT/RT3, all p values <0.001; 100 ± 0.8 vs. 56.7 ± 1.9 or 56.0 ± 3.2 % in SNU-AT/RT4, all p values <0.001; Fig. 4a). Likewise, cellular proliferation was also significantly suppressed (negative siRNA vs. LIN28B siRNA-1 or LIN28B siRNA-2: 100 ± 1.1 vs. 56.2 ± 5.2 or 58.0 ± 4.8 % in SNU-AT/RT3, all p values; 100 ± 1.0 vs. 58.0 ± 4.8 or 44.0 ± 4.3 % in SNU-AT/RT4, all p values; Fig. 4b).

To determine whether LIN28B affects cell cycle, we compared cells treated with negative and LIN28B siRNAs. We observed that the inhibition of LIN28B induced cell cycle arrest, particularly at the G1 phase (negative siRNA versus LIN28B siRNA-1 or LIN28B siRNA-2: 49.8 ± 2.7 vs. 63.7 ± 5.4 or 61.2 ± 2.1 % in SNU-AT/RT3, all p values <0.001; 52.3 ± 1.8 vs. 63.0 ± 4.4 or 61.5 ± 0.6 % in SNU-AT/RT4, all p values <0.001; Fig. 4c).

Next, we further examined cell migration after LIN28B knockdown. The results of the transwell migration assay showed that migration of AT/RT cells was significantly suppressed after LIN28B knockdown (comparison of migrated cells: negative siRNA vs. LIN28B siRNA-1 or LIN28B siRNA-2: 100 ± 7.2 vs. 30.2 ± 0.5 or 14.0 ± 2.3 % in SNU-AT/RT3, all p values <0.001; 100 ± 20.2 vs. 29.0 ± 9.0 or 17.6 ± 1.9 % in SNU-AT/RT4, all p values <0.001; Fig. 4d).

### Changes of gene expression by LIN28B knockdown

LIN28B is a pluripotency-related gene that governs early embryogenesis. We investigated the expression of pluripotency-related genes, such as SOX2, OCT4, C-MYC, NANOG, and KLF4, after LIN28B knockdown in primary cultured AT/RT cells. The expression of NCAM, a marker of early neurogenesis, and β-actin were measured for comparison.

**Fig. 5:**
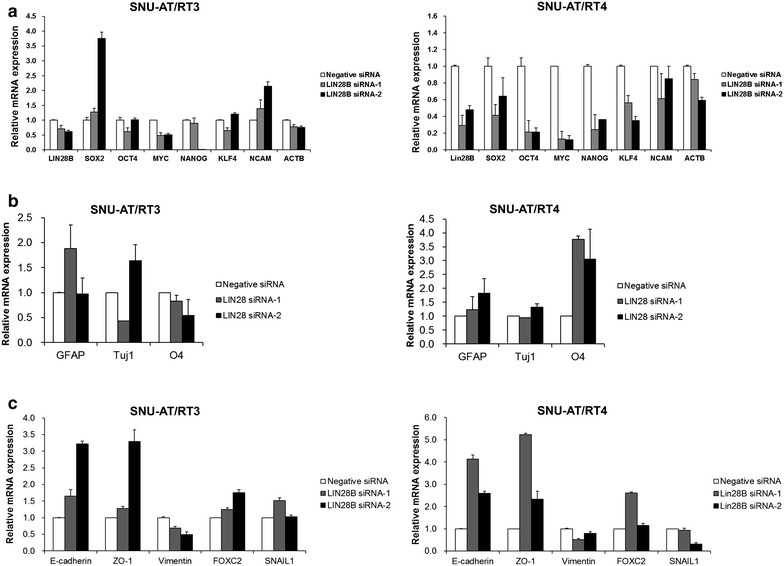
Regulation of pluripotent stem cell, differentiation and epithelial and mesenchymal cell-related gene expression through LIN28B knockdown. **a** After LIN28B knockdown, all pluripotent stem cell-related gene expression was reduced in SNU-AT/RT4, and SOX2 and NCAM was increased in SNU-AT/RT3. **b** The expression of GFAP, Tuj1 and O4 was variable in AT/RT cells. **c** The level of E-cadherin and ZO-1 was elevated and Vimentin was decreased in both AT/RT cell lines. *Error bars* represent ±SD

RT-qPCR analysis showed that expression of OCT4, MYC, NANOG, and KLF4 were decreased in SNU-AT/RT3and SNU-AT/RT4 (Fig. 5a). The pattern of SOX2 expression was variable, with an increase in SNU-AT/RT3 and a decrease in SNU-AT/RT4. In SNU-AT/RT3, the increased expression of SOX2 and NCAM were observed after LIN28 siRNA treatment. This increase likely reflects enhanced neuroglia differentiation, as SOX2 is also a marker of immature glia and NCAM is a marker of early neurogenesis. To determine whether the suppression of LIN28B and other pluripotency-related genes leads to cellular differentiation, we confirmed the mRNA expression of GFAP, TUJ1 and O4 after LIN28B suppression (Fig. 5b). Although the changes in expression did not convey a solid pattern, general increase in the expression of differentiation markers were observed.

### Regulation of epithelial and mesenchymal cell-related gene expression

The epithelial-mesenchymal transition (EMT) is associated with cancer cell migration and aggressiveness. After LIN28B knockdown, we examined the changes in EMT-related marker expression, including epithelial markers (E-cadherin and ZO-1) and a mesenchymal marker (Vimentin). We observed that E-cadherin and ZO-1 expression was increased and Vimentin expression was decreased in both AT/RT cell lines (Fig. 5c).

### Restoration of SMARCB1expression in AT/RT cells

**Fig. 6:**
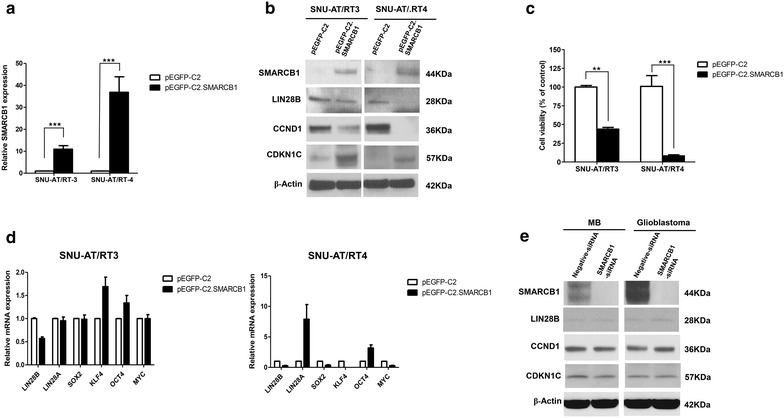
Overexpression of SMARCB1in AT/RT, MB and glioblastoma cells. **a** Transfection efficiency of SMARCB1 was confirmed using RT-qPCR at 48 h after pEGFP-C2.SMARCB1 transfection. **b** Transfection with pEGFP-C2.SMARCB1 decreased LIN28B and CCND1 expression and increased CDKN1C expression. **c** Cell viability was significantly diminished after the introduction of SMARCB1. **d** LIN28B was suppressed and OCT4 was increased after the restoration of SMARCB1 expression in all AT/RT cells. However, the differential expression of LIN28A, SOX2, KLF4 and MYC was detected in all AT/RT cells. **e** In MB and glioblastoma, the expression of LIN28B, CCND1 and CDKN1C was unchanged after SMARCB1 knockdown. **P < 0.01; ***P < 0.001. *Error bars* represent ±SD

To determine whether SMARCB1 regulates LIN28B expression, we transfected pEGFP-C2.SMARCB1 into primary cultured AT/RT cells (Fig. 6a). Subsequently, we performed an immunoblot analysis of LIN28B, CCND1 and CDKN1C.The decreased expression of LIN28B and CCND1 and increased expression of CDKN1C were observed (Fig. 6b). Transfection of pEGFP-C2.SMARCB1 reduced cell viability (pEGFP-C2 vs. pEGFP-C2.SMARCB1: 100 ± 2.1 vs. 43.8 ± 2.3 % in SNU-AT/RT3, p = 0.0019; 100 ± 14.4 vs. 8.1 ± 1.5 % in SNU-AT/RT4, p < 0.001; Fig. 6c). We further investigated the expression of pluripotency-related genes using RT-qPCR in 2 AT/RT cell lines and observed different expression levels of the genes. SMARCB1 expression significantly suppressed LIN28B gene expression in AT/RT cells (Fig. 6d). In SNU-AT/RT3 cells, KLF4 and OCT4 expression were increased, but the expression of LIN28A, SOX2 and MYC remained unchanged (Fig. 6d). In SNU-AT/RT-4 cells, LIN28A and OCT4 were increased, but SOX2, KLF4 and MYC expression was decreased (Fig. 6d). Interestingly, only OCT4 was elevated in both AT/RT cell lines.

Notably, the basal expression levels of SMARCB1 were low in SMARCB1-competent cancers, such as glioblastoma and medulloblastoma. The knockdown of SMARCB1 using specific siRNAs in these primary cell lines yielded little changes in the expression of LIN28B, CCND1 and CDKN1C (Fig. 6e).

## Discussion

AT/RT is one of the most aggressive and refractory cancers in humans. The most important characteristics of this disease are early-age onset and a typical genetic mutation. AT/RT predominantly develops during the infantile period, and the development of tumors in neonates is not uncommon [1]. Tumor development in neonates is a distinct feature of AT/RTs, as the peak age of diagnosis for patients with medulloblastoma is between 5 and 9 years old. The loss of SMARCB1 expression through genetic mutation/deletion is another distinctive feature of AT/RT [17]. This feature is highly specific for AT/RTs in pediatric neuro-oncology; the loss of SMARCB1 expression in immunohistochemistry strongly supports the diagnosis of AT/RT rather than medulloblastoma or choroid plexus carcinoma [18]. Intriguingly, a genome-wide analysis revealed that SMARCB1 aberration is the only recurrent mutation observed in AT/RT [19]. Indeed, AT/RT has the least number of gene mutations among human cancers [20] in contrast with the heavy mutation loads in adult-type cancers, such as glioblastomas [21]. Even medulloblastoma, which falls into the same group of embryonal brain tumor, shows the presence of ~10 recurrent genomic alteration per cancer [22]. For this reason, AT/RT is represented as a “true” embryonal tumor [23]. It is highly plausible that AT/RT has an activated embryonic/fetal gene expression program for which the loss of SMARCB1 in AT/RT leads to alterations in gene expression. LIN28 is a RNA-binding protein that regulates the function of the let-7 miRNA family. The balance of LIN28 and let-7 miRNA is important during embryonic development and for the maintenance of embryonic stem cells. LIN28 and let-7 miRNA constitute a reciprocal regulatory loop, and the loss of LIN28 expression leads to cellular differentiation. In contrast, LIN28 overexpression has been observed in many advanced human cancers and is associated with poor prognosis [9]. Specifically, the overexpression of LIN28B has been observed in central nervous system primitive neuroectodermal tumors (PNETs) and AT/RT.

Present study shows that the overexpression of LIN28B is more specific for AT/RT, and there is no significant difference in LIN28A expression between AT/RT and medulloblastoma tissues. Therefore, we focused on the role of LIN28B in AT/RT pathogenesis. The knockdown of LIN28B expression suppressed cell viability and proliferation, consistent with decreased CCND1 and enhanced CDKN1C expression. LIN28 is a pluripotency-related gene. Yu et al. [24] reprogrammed human somatic cells into induced pluripotent stem cell lines using OCT4, SOX2, NANOG, and LIN28. The knockdown of LIN28B in AT/RT cells decreased the expression of pluripotency-related factors and increased the expression of neuroglial differentiation markers. The variable responses of pluripotency- and differentiation-related genes reflect the heterogeneity and differentiation patterns of AT/RT tumors. AT/RT exhibits variable patterns of differentiation, including neuroglial, epithelial, and mesenchymal lineages, making histopathological diagnosis challenging. EMT is a phenomenon observed in embryogenesis, and this cellular transition is considered crucial in cancer cell migration and invasion. The knockdown of LIN28B significantly suppressed cell migration and the expression of mesenchymal phenotype (Vimentin and SNAIL1). Epithelial markers (E-cadherin and ZO-1) were increased, confirming a reversal of the EMT process (so-called MET phenomenon) [25]. The loss of SMARCB1 function is a key event in AT/RT pathogenesis. SMARCB1 is a component of the chromatic remodeling complex, which suppresses the expression of thousands of genes. The restoration of SMARCB1 expression in malignant rhabdoid tumor cells leads to cell cycle arrest and cellular senescence [26]. In the present study, we introduced SMARCB1 into AT/RT cell lines and observed the decreased expression of LIN28B, resulting in the disruption of CCND1-dependent cell proliferation. Interestingly, the knockdown of SMARCB1 in glioblastoma and medulloblastoma generated little effect on the LIN28B levels and the expression of CCND1 and CDKN1C. These results indicate that unlike truly embryonal tumors, such as AT/RT, SMARCB1-competent tumors are not dependent on the SMARB1-LIN28B pathway but rely on other signaling pathways for cellular survival and proliferation.

In conclusion, our results demonstrate that LIN28B might be regulated through SMARCB1, and the loss of SMARCB1 protein in AT/RT results in the unopposed expression of LIN28B and related oncogenes, such as CCND1, leading to tumorigenesis. Therefore, LIN28B may be a potential therapeutic target and biomarker of AT/RT for further clinical and translational studies.

## Additional file

10.1186/s12935-016-0307-4 mRNA/miRNA expression after LIN28 knockdown.
