# Supplementary material for: LIN28B is highly expressed in atypical teratoid/rhabdoid tumor (AT/RT) and suppressed through the restoration of SMARCB1
Source: Cancer Cell Int. 2016 Apr 18;16:32. doi: 10.1186/s12935-016-0307-4 (PMC4836086; doi:10.1186/s12935-016-0307-4)
Supplement: Supplementary file 1 — 10.1186/s12935-016-0307-4 mRNA/miRNA expression after LIN28 knockdown. [file 12935_2016_307_MOESM1_ESM.docx]

**Table S1. mRNA/miRNA expression after LIN28 knockdown**

| siRNAs | mRNAs/miRNAs | Negaitve siRNA | SNU-AT/RT3 | SNU-AT/RT4 |
| --- | --- | --- | --- | --- |
| LIN28BsiRNA-1 | LIN28B | 1.0 ± 0.01 | 0.6 ± 0.02, p=0.009 | 0.3 ± 0.02, p<0.001 |
| LIN28BsiRNA-1 | CCND1 | 1.0 ± 0.02 | 0.4 ± 0.08, p=0.012 | 0.5 ± 0.07, p=0.036 |
| LIN28BsiRNA-1 | CDKN1C | 1.0 ± 0.01 | 13.2 ± 1.02, p<0.001 | 2.8 ± 0.34, p=0.002 |
| LIN28BsiRNA-1 | C-MYC | 1.0 ± 0.03 | 0.5 ± 0.09, p<0.045 | 0.1 ± 0.08, p<0.001 |
| LIN28BsiRNA-1 | Pri-let-7g | 1.0 ± 0.01 | 1.2 ± 0.25, p=0.158 | 2.4 ± 0.35, p=0.002 |
| LIN28BsiRNA-1 | Mature let-7g | 1.0 ± 0.01 | 3.7 ± 1.06, p=0.001 | 4.3 ± 0.84, p<0.001 |
|  |  |  |  |  |
| LIN28BsiRNA-2 | LIN28B | 1.0 ± 0.01 | 0.7 ± 0.05, p=0.002 | 0.5 ± 0.03, p=0.020 |
| LIN28BsiRNA-2 | CCND1 | 1.0 ± 0.02 | 0.1 ± 0.07, p<0.001 | 0.8 ± 0.15, p=0.002 |
| LIN28BsiRNA-2 | CDKN1C | 1.0 ± 0.01 | 8.1 ± 0.58, p<0.001 | 3.5 ± 0.07, p=0.009 |
| LIN28BsiRNA-2 | C-MYC | 1.0 ± 0.03 | 0.5 ± 0.05, p<0.034 | 0.1 ± 0.01, p<0.001 |
| LIN28BsiRNA-2 | Pri-let-7g | 1.0 ± 0.01 | 1.6 ± 0.10, p=0.017 | 2.1 ± 0.06, p=0.004 |
| LIN28BsiRNA-2 | Mature let-7g | 1.0 ± 0.01 | 5.3 ± 5.15, p<0.001 | 7.0 ± 0.20, p<0.001 |

p value: negative siRNA versus SNU-AT/RT3 or SNU-AT/RT4
